# A rising star involved in tumour immunity: Lactylation

**DOI:** 10.1111/jcmm.70146

**Published:** 2024-10-17

**Authors:** Xu Zhang, Changming Liang, Chengwei Wu, Senlin Wan, Lishuai Xu, Song Wang, Jiawei Wang, Xiaoxu Huang, Li Xu

**Affiliations:** ^1^ Department of Gastrointestinal Surgery The First Affiliated Yijishan Hospital of Wannan Medical College Wuhu Anhui China; ^2^ Anhui Province Key Laboratory of Non‐coding RNA Basic and Clinical Transformation (Wannan Medical College) Wuhu China

**Keywords:** immunosuppression, lactate, lactylation, tumour

## Abstract

In recent years, continuous exploration worldwide has revealed that some metabolites produced during cellular and tissue metabolism can act as signalling molecules to exert different effects on the human body. These metabolites may act as cofactors for proteases or as post‐translational modifications linked to proteins. Lactate, a traditional metabolite, is found at high levels in the tumour microenvironment (TME). Many studies have shown that lactate influences tumorigenesis and development via different mechanisms, not only through the metabolic reprogramming of tumours but also through its significant impact on tumour immunity. Previously, tumour cells were reported to use glucose and glutamine to fuel lactate metabolism; however, lactate serves not only as an energy source for tumour cells but also as a precursor substance needed for the post‐translational modification of proteins. Recent studies identified a novel form of epigenetic modification, lactate‐mediated histone lysine lactylation (Kla) and demonstrated that histone lactylation directly stimulates chromatin after gene transcription; consequently, lactylation has become a popular research topic in recent years. This article focuses on the research progress and application prospects of lactylation in the context of tumour immunity.

## 1 INTRODUCTION

The immune system protects the body from damage caused by pathogens or tumour cells by detecting and eliminating abnormal cells.^1^ Therefore, the presence of tumour cells leads to the activation of intrinsic and adaptive immune responses, which are used to maintain homeostasis.^2^ However, tumour cells develop different mechanisms to evade the immune system, such as continuous remodelling at the genetic, epigenetic and metabolic levels to resist apoptosis and recognition by the immune system; tumour cells can also shift the phenotype and function of normal immune cells from a tumour‐suppressive phenotype to a tumour‐promoting phenotype. In addition, tumour cells facilitate the induction and recruitment of different immune cells and molecules to form an immunosuppressive environment that is conducive to tumour development.^3^ Moreover, metabolic alterations play important roles in the development, progression and maintenance of cancer.^4^ Lactate is considered an important tumour metabolite in cancer metabolic reprogramming, and high levels of lactate secreted by tumours promote the development of acidosis in the tumour environment^5^ and promote tumour development and immunosuppression.^6^ This effect may be due to increased levels of aerobic glycolysis in the tumour, which increases the concentrations of lactate in the tumour environment.^7^ Moreover, lactate may be used by tumour cells as an energy‐rich substrate, signalling molecule and important immunosuppressant.^8^

Protein post‐translational modifications (PTMs) are processes that alter the biochemical properties of a protein by adding a chemical group to one or more amino acid residues, thereby giving the precursor protein a specific function. Common post‐translational modifications of proteins include acetylation, ubiquitination, methylation, phosphorylation and glycosylation.^9^ In 2019, Zhang et al. identified a novel epigenetic modification on histone lysine residues called histone lactylation (abbreviated as histone Kla). In this study, the authors discovered a new function of lactate: it can regulate gene expression in macrophages through histone lactylation.^10^ In colon cancer, histone lactylation promotes Rubicon‐like autophagy enhancer (RUBCNL/Pacer) transcription. RUBCNL promotes autophagosome maturation through interaction with beclin 1(BECN1), leading to chemoresistance.^11^ H3K18la enhances immune escape in non‐small cell lung cancer (NSCLC) cells by activating the POM121/MYC/PD‐L1 pathway.^12^ H3K18la is enriched at the LCN2 promoter, and high LCN2 expression promotes bladder cancer progression.^13^ Additional studies have shown that increased levels of lactylation often suggest a poor prognosis.

Taken together, these findings highlight the strong potential for lactylation in tumours that can, among other effects, affect the immune system. Considering the recent discoveries on lactate‐mediated lactylation in the fields of tumour immunity and immunotherapy, we aimed to review the impact of lactate and lactylation on tumour immunity and the prospects for lactylation therapy. This review summarizes the current stage of research on lactylation, the findings of past research and future implications for these findings.

## 2 MOLECULAR MECHANISMS OF LACTYLATION IN DIFFERENT CONFIGURATIONS

**FIGURE 1:**
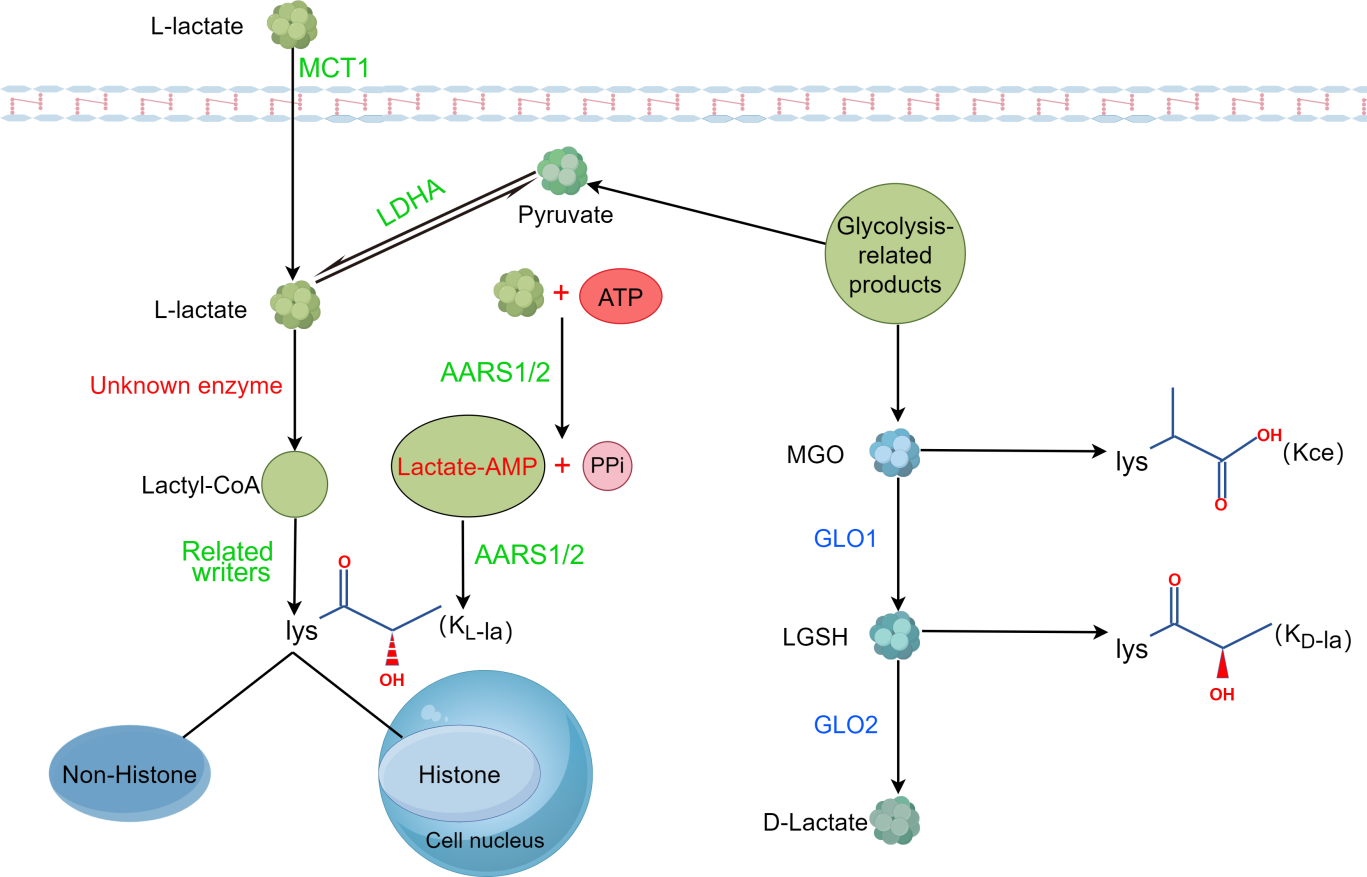
MCT1 transfers L‐lactate into the cell. The glycolysis byproduct pyruvate undergoes LDHA‐mediated conversion to produce L‐lactate. L‐lactate undergoes unknown enzymatic actions to synthesize lactyl‐CoA, which is involved in histone and nonhistone Kl‐la by the action of related writers. In addition, L‐lactate and ATP form lactate‐AMP and PPi in response to AARS1/2. Lactate‐AMP is covalently linked to lysines on target proteins. GLO1 conjugates MGO, a byproduct of glycolysis, to glutathione to form LGSH, which is then hydrolysed by GLO2 to produce D‐lactate and regenerate cellular glutathione. D‐la is formed by a noncatalytic reaction between LGSH and lysine residues of proteins. Kce is formed by a non‐catalytic reaction between MGO and lysine residues of proteins.

Lactate has two isomers, L‐lactate and D‐lactate. L‐lactate is the predominant isomer in the human body.^14^ L‐lactate is produced or scavenged by a reversible redox reaction catalysed by L‐lactate dehydrogenase (LDH). Lactate dehydrogenase A (LDHA) preferentially converts pyruvate to lactate.^15^ Lactate dehydrogenase A is a key enzyme in the metabolism of L‐lactate, and high levels of the LDHA isoform have been detected in muscle and tumours.^16^ Elevated levels of LDHA are a hallmark of many tumours, most of which are highly glycolytic.^17^, ^18^ On the other hand, D‐lactate is produced in the glyoxal pathway. Glyoxalase 1 (GLO1) conjugates methylglyoxal (MGO), a byproduct of glycolysis, to glutathione to form lactoylglutathione (LGSH), which is then hydrolysed by glyoxalase 2(GLO2) to produce D‐lactate and regenerate cellular glutathione.^19^, ^20^ Three different isomers of lactylation: the K_L‐la_ isomer is formed via the conversion of L‐lactate to lactyl‐coenzyme A (lactyl‐CoA), which is added to lysine residues via writers (acetyltransferase CBP/p300, KAT8, TIP60).^10^, ^21^, ^22^ The erasers of K_L‐la_ that have been identified are HDAC1‐3, SIRT2 and SIRT2, which mainly target non‐histone K_L‐la_.^23^, ^24^ Notably, the writers alanyl‐tRNA synthetase 1 (AARS1) and AARS2 transfer lactate to the lysine of target proteins by catalysing the ATP‐dependent formation of lactate‐AMP.^25^, ^26^, ^27^ On this basis, Junyi Ju et al. demonstrated that AARS1 fails to catalyse the onset of protein lactate modification even at 100‐fold higher physiological concentrations of lactyl‐CoA.^28^ K_D‐la_ is formed by a non‐catalytic reaction between LGSH and lysine residues of proteins.^19^ Kce is formed by a non‐catalytic reaction between MGO and lysine residues of proteins.^29^ Interestingly, Kla in histones is dominated by K_L‐la_ and the modification sites tend to be located in histone N‐terminal tails and gene promoters. This finding appears to be related to the fact that L‐lactate is the predominant form present in the human body. The source of lactyl‐CoA is currently controversial. Zhang et al. suggested that lactate is esterified to lactyl‐CoA by an as yet unidentified synthase.^10^ Marissa N. Trujillo et al. suggested that lactyl‐CoA may be derived from LGSH rather than through lactate and an unknown lactyl‐CoA synthetase.^20^ YdiF has been found to catalyse the formation of lactyl‐CoA in *Escherichia coli*. No reports have been published in relation to animal cells; therefore, the source of lactyl‐CoA production remains a major research question. The cellular levels of L‐lactate were much greater than those of MGO, LGSH, or D‐lactate. Although K_L‐la_ predominates in histone lactylation, the presence of structural isomers in non‐histone lactylation needs to be distinguished.^30^ Current studies have focused on K_L‐la_, probably because it plays a greater role in related diseases. Antibodies to differentiate structural isoforms already exist, which will help us to better differentiate isoform and carry out the next steps in our research (Figure 1).

## 3 LACTYLATION SHOWS STRONG POTENTIAL

Integrative lactylome and proteome analyses of tumours and adjacent livers revealed 9275 Kla sites, with 9256 sites on non‐histone proteins, suggesting that Kla is a pervasive modification within the cell involved in protein synthesis and transcriptional regulation. Kla may have an impact on a wide array of cellular events, such as gene expression, signalling and cellular morphology.^31^ The lactylation of proteins promotes microglia‐mediated tissue repair and induces collagen synthesis in fibroblasts to promote skin rejuvenation.^32^, ^33^ Lactylation is also involved in the regulation of atherosclerosis, sepsis‐associated injury, kidney fibrosis and many other diseases.^34^, ^35^, ^36^, ^37^ We focused on the role of lactylation in promoting malignant tumour progression, immunosuppression and chemotherapeutic resistance by affecting gene expression in tumours and immune cells.^38^ With increasing research worldwide, lactylation has shown strong potential in tumours. Studying the effects of lactylation on tumours and their crosstalk with other tumour regulatory mechanisms is important. These findings will improve our understanding of tumours and their treatment.

**FIGURE 2:**
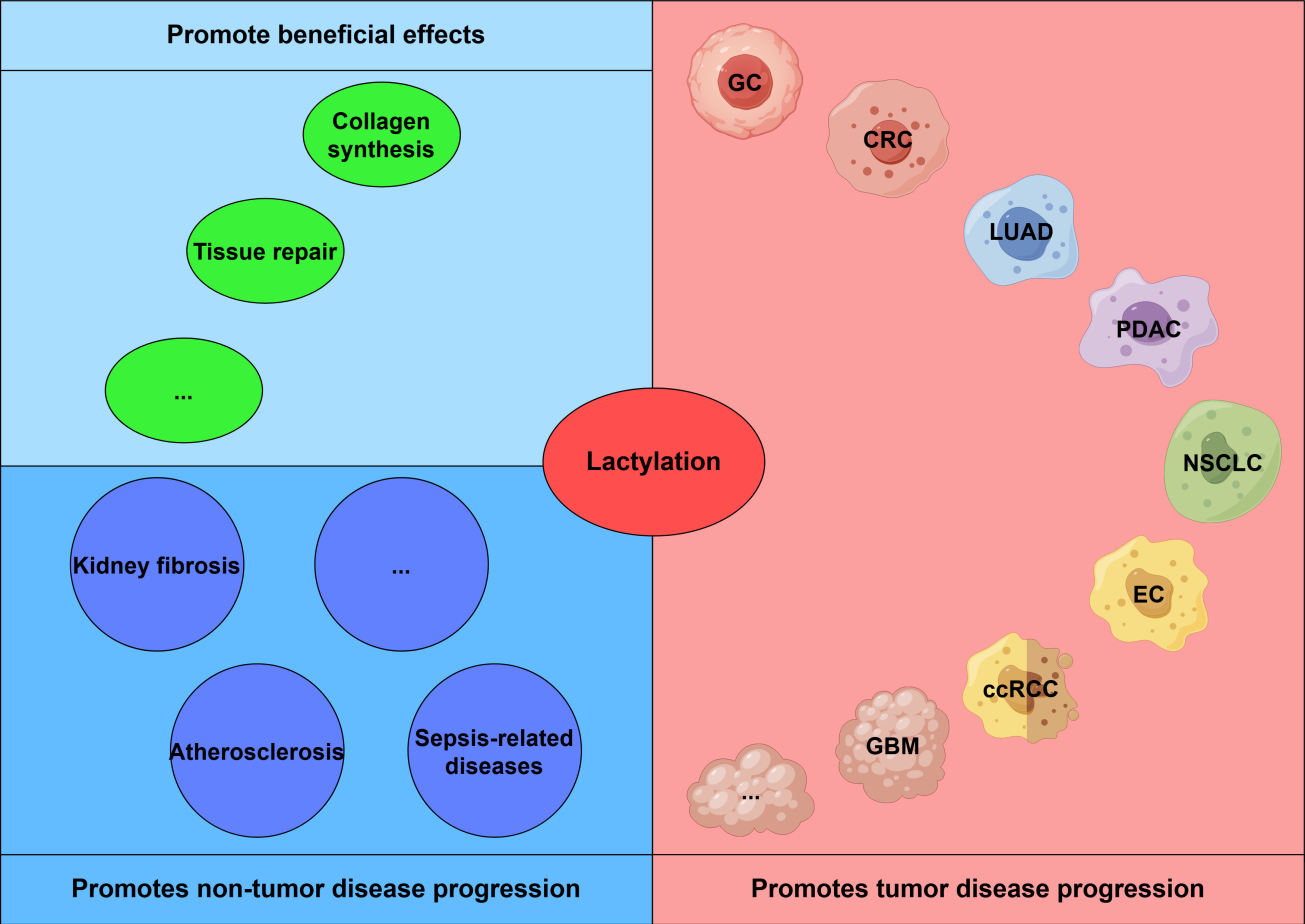
The lactylation is involved in tissue repair and induces fibroblasts to synthesize collagen, thus promoting skin rejuvenation. Lactylation is also involved in a variety of disease and tumour development processes.

Interestingly, similar to acetylation, butyrylation, crotonylation, etc., lactylation is mediated by lactate from glucose metabolism as a precursor substance. The known writers involved in lactylation share the same features as those involved in other PTMs.^39^ However, Junyi Ju and Zhi Zong et al. reported that AARS1 binds to lactate, catalyses the formation of lactate‐AMP, and then transfers lactate to lysine receptor residues. β‐Alanine disrupts the binding of lactate to AARS1.^25^, ^28^ Heyu Li and Yunzi Mao et al. reported that AARS2 is also involved in lactylation through this mechanism.^26^, ^27^ Considering the increase in endogenous lactate caused by elevated levels of aerobic glycolysis in tumours and the low detected levels of lactyl‐CoA,^40^ the possibility that tumours transfer lactate to lysine residues by forming lactate‐AMP via the non‐acyltransferase pathway is somewhat convincing. However, the current study is insufficient and further exploration is needed (Figure 2).

## 4 LACTYLATION MODULATES TUMOUR IMMUNITY

### 4.1 Lactylation regulates the tumour immune microenvironment (TIME)

Among bone marrow cells, tumour‐associated macrophages (TAMs), especially M2 macrophages, are associated with a poor prognosis.^41^ M1 macrophages have low arginase I (ARG1) levels, and arginine is metabolized by nitric oxide synthase to produce nitric oxide to kill pathogens, whereas M2 macrophages have high ARG1 levels and produce ornithine to promote wound healing.^42^ Treating bone marrow‐derived macrophages (BMDMs) with tumour cell‐derived lactate drives the M2‐like phenotypic profile of TAMs.^43^ Zhang et al. reported a positive correlation between Arg1 expression and histone lactylation levels in TAMs isolated from B16F10 melanoma and LLC1 lung tumours, demonstrating that histone lactylation activates M2‐like gene expression.^10^ This phenomenon was also demonstrated in PTEN/p53‐deficient prostate cancer cells, in which increased lactate production epigenetically reprogrammed TAMs through histone lactylation and polarized them to an immunosuppressive phenotype (M2/MHC‐II TAMs).^44^ Interestingly, lactate increases ARG1 expression in macrophages.^45^ Another study showed that lactate production by human cervical cancer cells results in macrophage polarization to an immunosuppressive phenotype.^46^

Another study identified two lactylation sites, K281R and K345R, in the zinc finger structural domain of methyltransferase like 3 (METTL3); these sites are required for the capture of target RNAs by METTL3. Tumour ‐derived lactate mediates METTL3 lactylation to increase the binding capacity of RNA, and METTL3 lactylation in TIMs promotes m6A‐mediated immunosuppression in the tumour microenvironment (TME).^47^ Signal transducer and activator of transcription 5 (STAT5) induces the accumulation of large amounts of lactate in AML cells by promoting glycolysis, which promotes dihydrolipoamide dehydrogenase (E3)‐binding protein (E3BP) nuclear translocation and increases H4K5la levels on the PD‐L1 promoter, which in turn induces PD‐L1 transcription.^48^ Monocarboxylate transporter protein 4 (MCT4)‐mediated lactate induces HIF1A histone lactylation in advanced prostate cancer (PC) cells, stabilizes HIF1α protein expression in a normoxic environment, enhances PD‐L1 transcription and promotes PC progression.^49^ Both lactate and LDHA in the TME can promote the protumorigenic activity of TAMs by promoting PDL‐1 expression.^50^ The occurrence of lactylation further enhances the immunosuppressive phenotype of the TME. Lactylation is further facilitated by the accumulation of large amounts of endogenous lactate as well as an increase in glycolytic activity. This finding provides insight into the infinite possibilities that exist in the regulation of the tumour immune microenvironment by lactylation.

### 4.2 Lactylation affects immune cells

**FIGURE 3:**
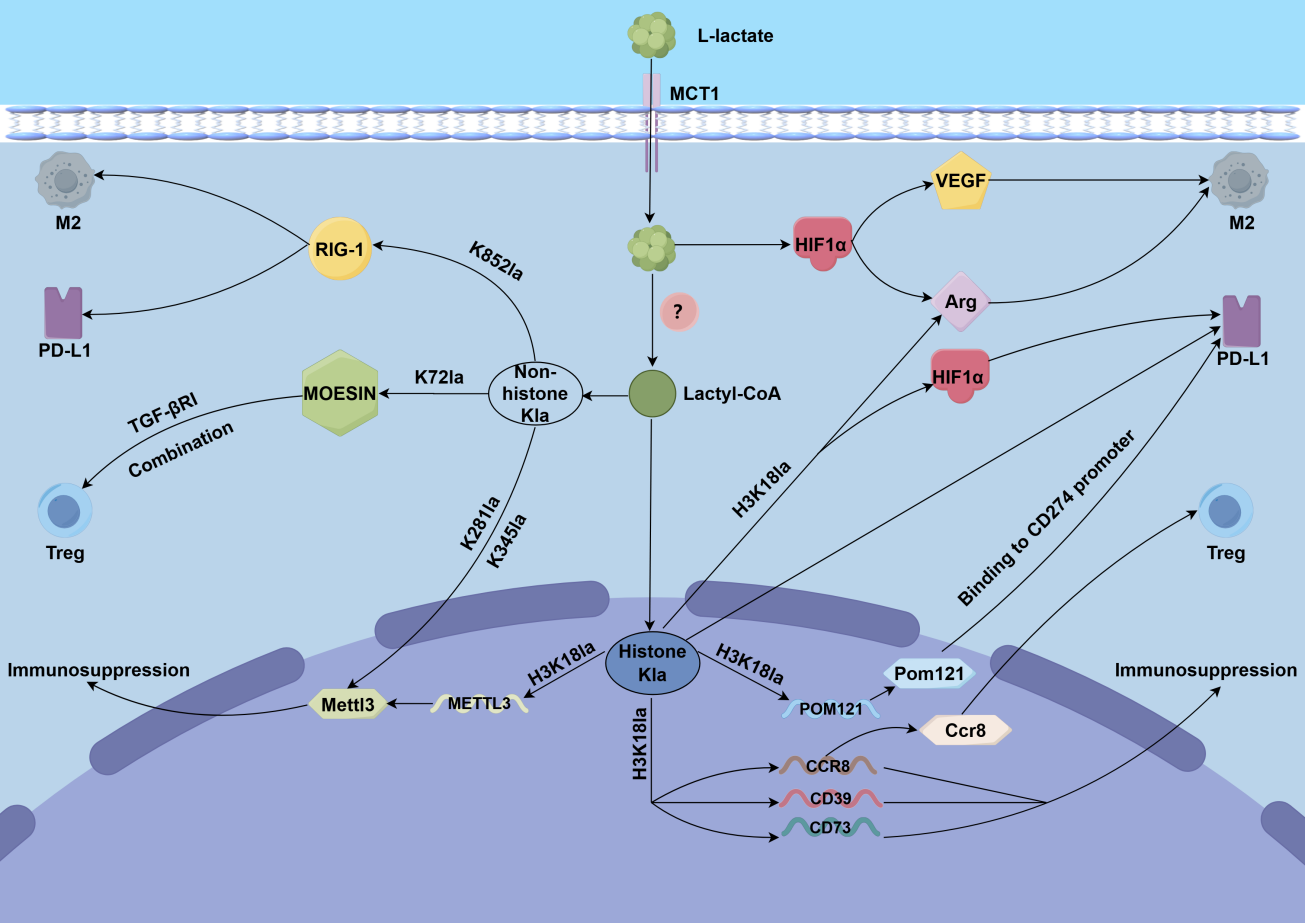
Lactylation is involved in the formation of the TIME and regulates immune cells.

Prior to the focus on lactate as a precursor substance for lactylation, lactate is known to act as a signal modulator that promotes tumour cell adhesion, migration and invasion.^51^ In addition, lactate is transferred into tumour tissues mainly through monocarboxylate transporter protein 1 (MCT1) and MCT4, which results in a low pH microenvironment. This acidic environment can lead to tumour progression and therapeutic resistance.^52^, ^53^ Lactate controls immune escape by activating GPR81 on stromal dendritic cells via the paracrine pathway.^54^ Tumour cells exhibit high glycolytic activity, and tumour ‐derived lactate can promote the development of MDSCs.^55^ Lactate suppresses T‐cell autoimmunity by activating hypoxia‐inducible factor‐1 alpha (HIF‐1α)/NDUFA4L2 signalling in DCs.^56^ Lactate in the TME inhibits IFN‐γ and IL‐4 production by NKT cells; a low extracellular pH alone can induce NKT cell dysfunction, and the acidic microenvironment of the tumour may interfere with NKT cell function through metabolic control.^57^, ^58^ Regulatory T (Treg) cells play a crucial role in maintaining the immunosuppressive TME and are a major barrier to cancer immunity.^59^ The presence of lactate favours iTreg cells.^60^ Treg cells can maintain their inhibitory capacity through the use of lactate in the TME.^61^ In one study, lactate in the TME induced histone H3K18la, which increased the activity of the CD39, CD73 and CCR8 gene promoters and promoted immunosuppression. In addition, upregulation of the CCR8 pathway activated Treg cells, enhancing immunosuppression and disturbing the Th17/Treg balance.^62^ High levels of H3K18la have been detected in Th3 and Th1 cells as well as in Treg cells, which suggests that histone lactylation is a widespread PTM in activated T cells.^63^ MOESIN is indispensable for efficient transforming growth factor beta (TGF‐β) signalling.^64^ The lactylation of MOESIN at the Lys72 locus enhances TGF‐β signalling in Treg cells via TGF‐βRI, which promotes the production and enhances the function of Treg cells, thereby facilitating the immune escape of tumour cells.^60^ In the microbiome, retinoic acid‐inducible gene 1 (RIG‐I) lactylation inhibits CD8+ T‐cell activation via mTOR and enhances immunosuppression in Treg cells by promoting PD‐1 expression.^65^ These findings suggest that lactylation influences the role of immune cells in tumour cell surveillance and is inextricably linked to high lactate levels. The mechanism by which high levels of lactate affect immune cells through lactylation should also be further explored. In the context of tumour immunosuppression, studies of these mechanisms should focus on factors that play a role in elevated levels of lactate in tumours, as well as the key genes or proteins involved in mechanisms that promote tumour glycolysis (Figure 3).

## 5 LACTYLATION REGULATES TUMOUR GLYCOLYTIC ACTIVITY

The activation of receptor tyrosine kinases (RTKs) has been reported to be correlated with increased glycolysis.^66^ Platelet‐derived growth factor receptor beta (PDGFRβ), a transmembrane receptor tyrosine kinase, was identified as a key target gene for histone lactylation in clear cell renal cell carcinoma (ccRCC).^67^ Recent results revealed that stimulation of PDGFβ signalling in ccRCC cells promoted glycolysis, lactate production and histone lactylation, which in turn activated PDGFRβ transcription, suggesting that there is a positive feedback loop between histone lactylation and PDGFRβ signalling, resulting in a vicious cycle that further accelerates ccRCC progression.^68^ Increased levels of H3K18la in the promoter region in pancreatic adenocarcinoma (PDAC) promote the transcription of TTK protein kinase (TTK) and BUB1 mitotic checkpoint serine/threonine kinase B (BUB1B) to promote tumour progression. Glycolysis inhibitors reduce histone lactylation and inhibit PDAC cell proliferation and migration.^69^ In endometrial cancer (EC), elevated levels of H3k18la in the ubiquitin‐specific peptidase 39 (USP39) promoter region increase its transcriptional level to promote EC progression. In addition, USP39 enhances PI3K/AKT and HIF‐1α‐mediated glycolysis in EC cells to further promote EC progression.^70^ In oesophageal carcinoma cells, hypoxia induces lactylation of the Axin1 protein at K147 and promotes ubiquitination of the Axin1 protein, leading to decreased protein stability, increased glycolysis and promotion of oesophageal carcinoma cells.^71^

In conclusion, some types of histone lactylation further promote glycolysis by increasing the transcription of related genes, thus increasing the level of lactate and creating a vicious cycle. Non‐histone lactylation may increase tumour glycolysis by affecting downstream molecules. Glycolysis inhibitors and lactate inhibitors can downregulate the level of lactylation and thus inhibit the malignant progression of tumours. Therefore, studying the relationship between lactylation and tumour glycolysis may be a key step in improving tumour therapy.

## 6 PERSPECTIVES ON LACTYLATION IN TUMOUR THERAPY

### 6.1 Lactylation is involved in tumour drug resistance

Lactylation sites are widely distributed within cells; however, the presence of lactylation sites varies among different cells.^72^ Bevacizumab treatment exacerbates glycolysis in hypoxic cancer cells, thereby resulting in the production of lactate. Higher levels of H3K18la subsequently increase RUBCNL transcription, thereby contributing to colorectal cancer cell survival and treatment resistance. Compared with bevacizumab treatment alone, the combination of bevacizumab with the inhibition of histone lactylation and autophagy inhibited proliferation and increased apoptosis.^11^ High levels of lactylation have been observed in recurrent glioblastoma (GBM) and temozolomide (TMZ)‐resistant cells. Upregulated H3K9la activates LUC7L2 transcription, and LUC7L2 mediates the retention of MLH1 intron 7 to reduce MLH1 expression, inhibit MMR (mismatch repair) and ultimately promote TMZ resistance in GBM.^73^ In NSCLC, the lactylation of apolipoprotein C‐II (APOC2) at the K70 site stabilizes protein levels by inhibiting its ubiquitination. K70 lactylation of APOC2 induces elevated free fatty acid (FFA) levels, promoting Treg cell enhancement and immunotherapy resistance. An antibody specific for the K70 lactylation site of APOC2 has been developed and is positively correlated with immunotherapy resistance in NSCLC.^74^ These findings demonstrate the feasibility of combination therapy through the development of antibodies specific to lactylation sites. However, drug resistance therapies for the development of histone lactylation site‐specific antibodies have not been investigated, perhaps because blocking lactylation on the histone in question without affecting other normal cellular functions is difficult. The effects of utilizing a wide range of histone lactylation inhibitors on other cellular functions are not known.

### 6.2 The potential of lactylation as a predictive biomarker for various types of cancer

Demethylzeylasteral (DML) can be targeted to treat hepatocellular carcinoma (HCC); specifically, it targets lactate to regulate H3 Kla. Mechanistically, DML inhibits H3Kla through H3K9la and H3K56la, which suppresses the tumorigenic properties of liver cancer stem cells (LCSCs).^75^ Later, Wu et al. suggested that histone lactylation was associated with the immune microenvironment of HCC and could be considered an independent biomarker. Comprehensive analysis of histone lactylation ‐specific genes revealed that NR6A1, OSBP2 and UNC119B could promote HCC progression and lead to treatment resistance. In addition, NR6A1, OSBP2 and UNC119B may induce the activation of pathways associated with HCC progression, including the WNT, MAPK, MTOR and NOTCH signalling pathways. These findings suggest that NR6A1, OSBP2 and UNC119B may be novel therapeutic targets for HCC immunotherapy and chemotherapy.^76^ Therefore, studies on Kla, a new therapeutic target in HCC, may contribute to the treatment of HCC.

In gastric cancer (GC), high Kla levels are associated with poorly differentiated tumours, lymph node metastasis and poor overall survival in patients.^77^ A lactylation score model that can be used to predict malignant progression and immune escape in GC has been established, and this model can also predict the therapeutic response of GC to immune checkpoint inhibitors (ICIs). The lactate‐related genes in the constructed model are also expected to be potential therapeutic targets and diagnostic markers for GC.^78^ The lactylation of METTL16 provides a potential target for assessing the efficacy of copper ion carrier drugs in GC.^23^ In addition, histone lactylation promotes breast cancer progression by regulating c‐Myc expression in an H3K18la‐dependent manner.^79^ In addition to the previous finding that histone lactylation plays a role in melanoma development,^80^ interest in the effects of lactylation on tumours and their response to treatment has increased. Histone lactylation ‐related gene analyses have been performed for breast cancer and melanoma patients and revealed identify lactylation‐related targets to guide immunotherapy for related tumours.^81^, ^82^ In PDAC, the lactylation model was used to predict the immune status and therapeutic response of PDAC patients, providing new strategic directions and antitumour immunotherapy options for PDAC patients. A link between SLC16A1 and lactylation scores was also identified that can be used to guide research.^83^ The prediction of lactylation sites can help us conduct corresponding research and discover new research ideas. The construction of lactylation scoring models in different tumours through research is beneficial for early diagnosis and for guiding the use of medication. This topic warrants more in‐depth research.

### 6.3 Targeting molecules upstream of lactylation

Histone lactylation is sensitive to lactate levels, and inhibition of glycolysis impairs lactate production, followed by a decrease in histone lactylation levels, whereas elevated lactate production increases Kla levels.^10^ Oxalate was found to inhibit lactate production and downregulate CD39, CD73 and CCR8 gene promoter activity by decreasing histone H3K18 lactylation. Oxalate also alters the immunosuppressive TME and promotes immune activation, suggesting that enhancing chimeric antigen receptor (CAR)‐T‐cell function may be a potential strategy for GBM treatment.^62^ Furthermore, ALDOB‐mediated lactate production increases carcinoembryonic antigen‐related cell adhesion molecule 6 (CEACAM6) protein stability by inducing Kla, which promotes cell proliferation and 5‐FU chemoresistance in colorectal cancer (CRC) cells.^84^ These findings suggest that lactate‐induced lactylation may be an important factor in tumour immunotherapy resistance.

Enzymes related to lactate production and transport, such as MCTs and LDHA, have long been of interest. MCT1 was previously shown to be a key regulator of lactate exchange between tumour cells and MCT1 is expressed in a range of primary human tumours. Lactate produced during the Warburg effect stimulates HIF‐1α expression, thereby exacerbating the malignant phenotype of cancer. Pierre Sonveaux et al. reported that MCT1 blockers can act as bona fide HIF‐1α inhibitors with antiangiogenic effects, and MCT1 inhibition has been identified as a therapeutic modality that exhibits both antimetabolic and antiangiogenic activity.^85^ However, a preclinical study, revealed that combining the MCT1 inhibitor AZD3965 with anti‐PD‐1 therapies reduced lactate release into the TME and increased the efficacy of tumour immunotherapy.^86^ In addition, the combination of anti‐PD‐1 agents and LDH inhibitors had more potent antitumour effects than did anti‐PD‐1 agents alone.^60^ Inhibition of MCT4 reverses lactate‐driven immunosuppression, and blocking MCT4 increases the efficacy of immune checkpoint blockade in vivo.^87^ Therefore, the inhibition of endogenous lactate production and transport‐related enzymes, such as MCTs and LDHA, is useful for tumour treatment.

**FIGURE 4:**
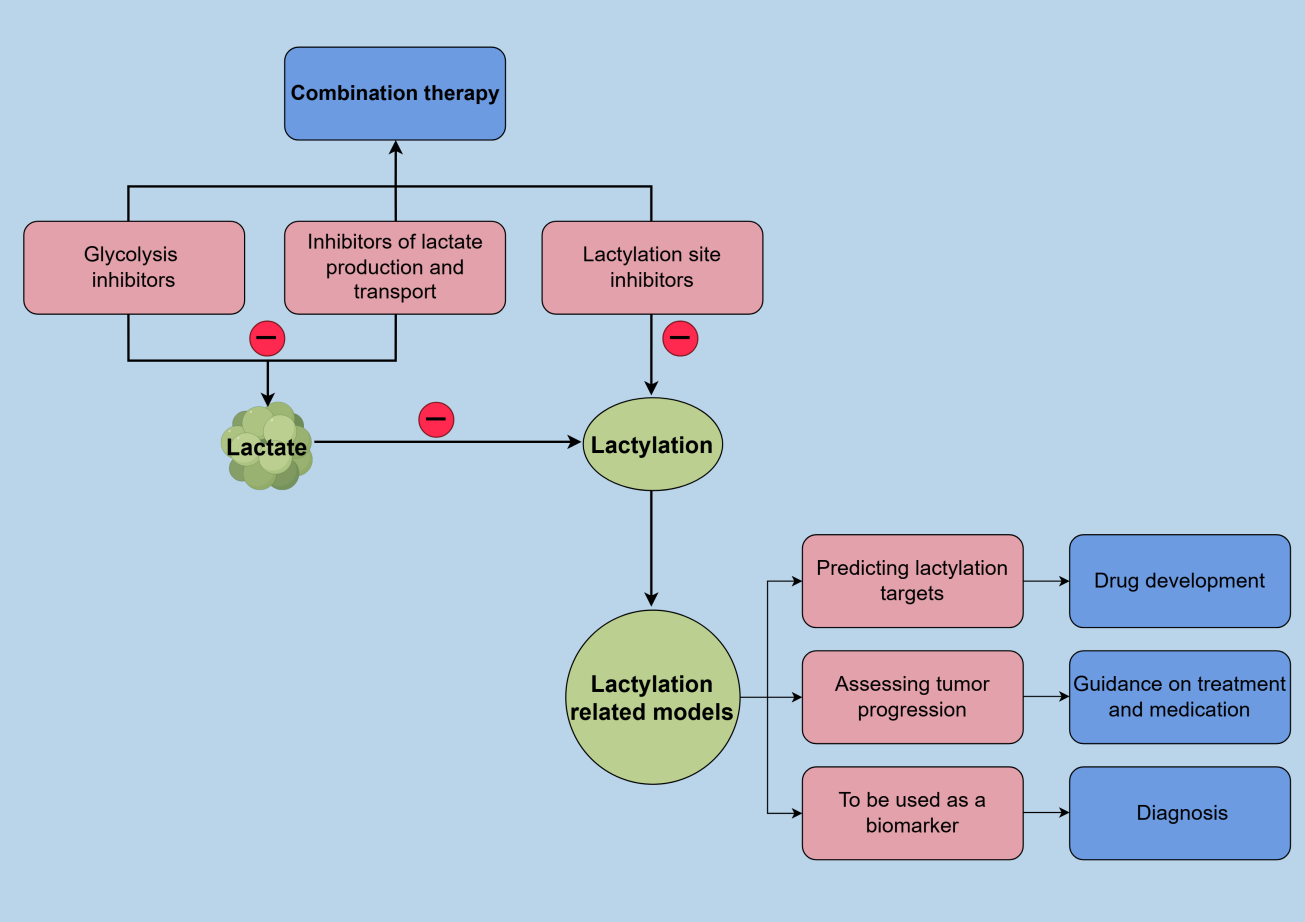
Inhibitors that target lactylation upstream and lactylation sites can be used in combination therapy for tumour immunity. Lactylation model constructs can be used to study tumour‐related mechanisms, guide therapy and early diagnosis.

In conclusion, lactylation sites are abundant on histones and non‐histone proteins in tumour cells, and safer and more effective therapeutic targets for the treatment of tumours can be identified by exploring the mechanism of action of Kla and its regulatory sites. Additionally, these studies could reveal new directions for immunotherapy combination strategies. Furthermore, decreasing the level of lactate in tumours, which also inhibits lactylation and impairs lactate homeostasis in the TME, is also a promising cancer treatment option and has been implemented in several preclinical and clinical trials. Therefore, it is also essential to establish synergistic effects between lactate inhibitors and other adjuvant therapies (Figure 4).

## 7 CONCLUSIONS

The development of Kla seems to be accompanied by elevated levels of lactate, which plays an important role as a precursor substance for Kla. Interestingly, tumour development enhances glycolytic activity in different ways,^48^, ^68^, ^88^, ^89^ which results in high levels of lactate, thereby increasing the level of lactylation in tumour cells, which in turn affects tumour cell metabolism, immunity, etc. Increased lactylation of some proteins increases glycolytic activity thus creating a positive feedback loop. Interestingly, the effects of lactylation on tumours seem to be beneficial to tumorigenesis and development; almost no inhibitory effects of lactylation on tumour growth, migration, or invasion have been observed; and whether there is an inhibitory phenomenon remains to be further investigated.

Endogenous lactate production is a key determinant of histone lactylation levels, and exogenous lactate also contributes to histone lactylation. Increased levels of lactate and LDHA in tumours seem to indicate elevated levels of lactylation, and in experiments where lactate production inhibitors were used, lactylation levels were decreased. Currently, most research on lactylation has focused on its downstream effects. In order to fully understand the complex conditions that lead to lactylation, more studies targeting its upstream regions are needed. Experimentally, the lactylation level and malignant behaviour of tumours were suppressed after treatment with glycolytic enzyme inhibitors and inhibitors of lactate production. These findings suggest that lactate is a precursor substance for lactylation. Currently, however, the key topic in lactylation is the ability to unravel the role of lactoyl‐CoA and the enzymes that produce it. This information will be useful for better understanding the source of lactylation and guiding the treatment of related diseases.

In addition, in terms of tumour immunotherapy, several key lactylation sites have been detected in tumours and in the TME. These sites can recruit tumour‐associated immune cells, remodel the microenvironment and cooperate with other epigenetic modifications to promote tumorigenesis and development, or even directly affect the expression of genes involved in key tumour‐associated pathways. Therefore, we can enhance immunotherapy by investigating the efficacy of combination immunotherapy with specific antibodies targeting the relevant lactylation sites. With the establishment of lactylation models for various types of tumours, we believe that research on tumour immunotherapy targeting lactylation sites will yield more and better options for cancer treatment in the future.

## AUTHOR CONTRIBUTIONS

**Xu Zhang:** Conceptualization (equal); supervision (equal); writing – original draft (equal); writing – review and editing (equal). **Changming Liang:** Conceptualization (equal); writing – original draft (equal). **Chengwei Wu:** Conceptualization (equal); writing – original draft (equal); writing – review and editing (equal). **Senlin Wan:** Conceptualization (equal); supervision (equal); writing – review and editing (equal). **Lishuai Xu:** Supervision (equal); writing – review and editing (equal). **Song Wang:** Conceptualization (equal); writing – original draft (equal). **Jiawei Wang:** Project administration (equal); supervision (equal); writing – review and editing (equal). **Xiaoxu Huang:** Writing – review and editing (equal). **Li Xu:** Writing – review and editing (equal).

## FUNDING INFORMATION

This work was supported by the National Natural Science Foundation of China (82372707, 81902515);Natural Science Research Project of Higher Education in Anhui Province (2023AH051771, KJ2021A0857, 2023AH040254); Anhui Provincial Health Commission Provincial Financial Key Projects(AHWJ2023A10126), Wuhu Science and Technology Program (2022cg27).

## CONFLICT OF INTEREST STATEMENT

The authors declare that the research was conducted in the absence of any commercial or financial relationships that could be construed as a potential conflict of interest.

## Data Availability

Data sharing not applicable to this article as no datasets were generated or analysed during the current study.
